# Dissecting the integrative antioxidant and redox systems in plant mitochondria. Effect of stress and *S*-nitrosylation

**DOI:** 10.3389/fpls.2013.00460

**Published:** 2013-11-28

**Authors:** Juan J. Lázaro, Ana Jiménez, Daymi Camejo, Iván Iglesias-Baena, María del Carmen Martí, Alfonso Lázaro-Payo, Sergio Barranco-Medina, Francisca Sevilla

**Affiliations:** ^1^Department of Biochemistry and Cellular and Molecular Biology of Plants, Estación Experimental del Zaidín, Consejo Superior de Investigaciones CientíficasGranada, Spain; ^2^Department of Stress Biology and Plant Pathology, Centro de Edafología y Biología Aplicada del Segura, Consejo Superior de Investigaciones CientíficasMurcia, Spain

**Keywords:** abiotic stress, ascorbate-glutathione cycle, mitochondria, peroxiredoxin, signaling, *S*-nitrosylation, sulfiredoxin, thioredoxin

## Abstract

Mitochondrial respiration provides the energy needed to drive metabolic and transport processes in cells. Mitochondria are a significant site of reactive oxygen species (ROS) production in plant cells, and redox-system components obey fine regulation mechanisms that are essential in protecting the mitochondrial integrity. In addition to ROS, there are compelling indications that nitric oxide can be generated in this organelle by both reductive and oxidative pathways. ROS and reactive nitrogen species play a key role in signaling but they can also be deleterious via oxidation of macromolecules. The high production of ROS obligates mitochondria to be provided with a set of ROS scavenging mechanisms. The first line of mitochondrial antioxidants is composed of superoxide dismutase and the enzymes of the ascorbate-glutathione cycle, which are not only able to scavenge ROS but also to repair cell damage and possibly serve as redox sensors. The dithiol-disulfide exchanges form independent signaling nodes and act as antioxidant defense mechanisms as well as sensor proteins modulating redox signaling during development and stress adaptation. The presence of thioredoxin (Trx), peroxiredoxin (Prx) and sulfiredoxin (Srx) in the mitochondria has been recently reported. Cumulative results obtained from studies in salt stress models have demonstrated that these redox proteins play a significant role in the establishment of salt tolerance. The Trx/Prx/Srx system may be subjected to a fine regulated mechanism involving post-translational modifications, among which *S*-glutathionylation and *S*-nitrosylation seem to exhibit a critical role that is just beginning to be understood. This review summarizes our current knowledge in antioxidative systems in plant mitochondria, their interrelationships, mechanisms of compensation and some unresolved questions, with special focus on their response to abiotic stress.

## INTRODUCTION

Plant mitochondria host some of the most important biological processes, i.e, oxidative phosphorylation, citric acid cycle and fatty acid oxidation. Based on their physiological relevance, mitochondria are involved in underpinning cellular proliferation, plant growth, development and death (Millar et al., 2011). Although chloroplasts and peroxisomes are the major ROS producers in plant cells under light periods (Foyer and Noctor, 2003), mitochondrial metabolism significantly accounts for the total ROS generation (Noctor et al., 2007). Overall, complexes I and III of the electron transport chain (ETC) are the main sites of ROS production and about 1–5% of the total consumed oxygen is converted into hydrogen peroxide (H_2_O_2_; Moller, 2001).

Initially, mitochondrial ROS were considered as an undesirable by product with deleterious effects. Higher ROS amounts resulting from uncontrolled ROS generation can cause oxidative stress by damaging cellular components and affecting organelle integrity. A growing number of publications now recognize the implication of ROS in many other cellular processes, including its proposed role as signaling molecules under oxidative conditions (Dat et al., 2000; Mittler et al., 2011). The condition of signaling molecules implies a tight control of ROS-antioxidants’ interplay in the different cell compartments, and the activation of signaling pathways by ROS responsive regulatory genes has been suggested as contributing to plant tolerance toward different stresses (Schwarzländer and Finkemeier, 2013). Therefore, the response of plants to ROS is dose dependent (Veal et al., 2007). Under stress conditions, the presence of ROS is not always a symptom of cellular dysfunction, but rather a signal to modulate transduction pathways through mitogen-activated protein kinases (MAPK) and transcription factors (Jaspers and Kangasjärvi, 2010). In mammals, this signaling process is present in several diseases and shows the crosstalk between multiple transcription factors and the redox-regulating protein Trx (Burke-Gaffney et al., 2005). In plants, a much less studied system, the involvement of Trx in redox signaling is being considered (Zaffagnini et al., 2012b).

Besides ROS, plant mitochondria have also emerged as an important site for nitric oxide production by two main pathways: a mitochondrial nitrite reducing activity whose site of NO^•^ generation remains uncertain (Planchet et al., 2005), and the oxidation of L-arginine by an elusive nitric oxide synthase (NOS; Guo and Crawford, 2005). Formation of ROS in junction with NO^•^ may present a danger in the mitochondria. To maintain the cellular redox homeostasis and avoid an oxidative stress that could cause molecular damage, plant mitochondria possess a set of antioxidant enzymes such as manganese superoxide dismutase (Mn-SOD), enzymes of the ascorbate-glutathione cycle and enzymes of the Trx/Prx/Srx system (Sevilla et al., 1982; Jiménez et al., 1997; Barranco-Medina et al., 2008b). These antioxidant scavengers respond to the stress situations (Martí et al., 2011) by regulating the level of ROS and modulating the redox signaling.

Along with ROS, reactive nitrogen species (RNS) are critical factors in signaling, by working as second messengers. The signaling process can be indirectly exerted by molecules that have suffered the oxidative damage by a reversible change in the redox state. Post-translational modifications (PTMs) of redox cysteine residues of targets proteins constitute a secondary mitochondrial retrograde regulation (MRR) and can modulate ROS and RNS signaling (Hartl and Finkemeier, 2012). Among them, *S*-glutathionylation and *S*-nitrosylation have emerged as novel regulators in cell signaling and response to stress conditions (Zaffagnini et al., 2012a; Camejo et al., 2013a). Protein oligomerization and reversible overoxidation of cysteine residues add a further step into the redox regulation (Barranco-Medina et al., 2009; Iglesias-Baena et al., 2010).

In this work we dissect the different aspects of the redox regulation of plant mitochondria, with special emphasis on the ascorbate-glutathione cycle and Trx/Prx/Srx system under stress.

## MITOCHONDRIA ARE ESSENTIAL SOURCES OF ROS AND RNS

Mitochondria are highly dynamic, metabolically active cell organelles. From a functional point of view, ETC in plant mitochondria differs from its animal counterpart in two additional pathways: alternative NAD(P)H dehydrogenases (type II NDH) and alternative oxidase (AOX). Both of these non-proton-pumping pathways could function as “safety valves” to limit ROS production by maintaning the ETC relatively oxidized (Moller, 2001; Rasmusson and Wallström, 2010; Millar et al., 2011). Plants ETC consists of four main complexes, some of them organized into supracomplexes (Dudkina et al., 2006). Supplementary to the NADH dehydrogenase, complex I and the flavoprotein complex II, the inner mitochondrial membrane contain type II NDH that bypass complex I and supply electrons to the ubiquinone pool and do not contribute to the generation of the proton motive force needed for ATP synthesis. Besides the usual cytochrome c oxidase (complex IV), a non-phosphorylating AOX is present. This enzyme bypasses the electron flow from complex III and IV, coupling the oxidation of ubiquinol with the reduction of oxygen to water, dissipating the energy as heat and lowering the ADP/O ratio. Shunting electrons through this pathway is important in energy-rich plants cells for primary and secondary metabolism, as well as for oxidation of excess carbohydrate (Rasmusson and Wallström, 2010). The expression of AOX and type II NDH, both of nuclear encoding, is increased during ETC inhibition by mitochondria to nucleus signaling (Van Aken et al., 2009a; Hartl and Finkemeier, 2012; Leister, 2012). In this process, organellar redox state and ROS metabolism have been poproposed as sources for retrograde signals which could trigger gene expression responses and provide a metabolic flexibility which, during stress conditions, play an important role in the acclimation of plants (Rhoads and Subbaiah, 2007; Woodson and Chory, 2008)

### ROS PRODUCTION

A key feature of mitochondrial biochemistry is the unavoidable production of ROS, with complex I and complex III being the major sites (Noctor et al., 2007). Under specific conditions ROS may be produced at complex II site, in the course of reverse electron transport (Turrens, 2003). ROS production is enhanced under conditions of high matrix NADH^+^/NAD. On the other hand, increased membrane potential correlates with more highly reduced ETC components, so raising the probability of single electron leak to oxygen and of $O_{2}^{\cdot -}$. This superoxide can, in turn, act as substrate for the generation of secondary ROS such as H_2_O_2_ and hydroxyl radical (^•^OH). The magnitude of membrane potential is dependent on the activity of the energy-dissipating systems, and on the oxidative phosphorylation. Hence, when ADP is being actively phosphorylated, membrane potential and ROS are lower than when ADP is limiting. Increased energy dissipation can similarly be achieved by artificial uncouplers, uncoupling proteins (UCPs; Moller, 2001; Finkel, 2011; Collins et al., 2012) and by the plant mitochondria potassium channel (PmitoK_ATP_) which can be stress-activated through several mechanisms, including activation by ROS, so indicating the fine regulation of this biochemical pathway. Dissipation of membrane potential directly by these components may be important in tissues with low AOX expression and/or activities (Trono et al., 2004). Similarly, in mammalian, H_2_O_2_ treatment of myoblast and cardiomyocyte mouse cells, increased the expression of the transcription factor Nrf2 that promoted the expression of the UCP, UCP3 decreasing ROS production and preventing cell death (Anedda et al., 2013).

Reactive oxygen species accumulation in mitochondria could also be influenced by PTM of respiratory complexes (Taylor et al., 2003; Beer et al., 2004), activity of alternative NADPH dehydrogenases (Rasmusson and Wallström, 2010) and modification of antioxidant systems and oxygen concentration (Jiménez et al., 1998). The relative importance of the different factors could be tissue specific (Noctor et al., 2007).

### NO^•^ PRODUCTION

In plants, two major enzymatic pathways are proposed to participate in NO^•^ formation: oxidation of L-arginine to L-citruline by a NOS like enzyme and reduction of nitrite to NO^•^ by a nitrate reductase (NR; Neill et al., 2003; Fröhlich and Durner, 2011; Gupta et al., 2011). In the past decade, the presence of NOS-like activity in plant peroxisomes was demonstrated. However, the characterization of such an enzyme is unresolved (del Río et al., 2002). To date, in contrast with mammalian tissue, the production of NO^•^ by a NOS-like enzyme in plant mitochondria remains elusive (Gupta and Kaiser, 2010). The reduction of nitrite to NO^•^ by the mitochondrial ETC contributes to ATP production under hypoxic conditions. NO^•^ production by a mitochondrial nitrite reducing activity has yet been detected in different photosynthetic sources and mitochondria isolated from roots of diverse plants species. These activities depend on the expression and/or activity of NR, since this enzyme is the main source of nitrite in plants (Wulff et al., 2009). Pharmacological evidences based on inhibitor sensitivity, suggests that complex III, cytochromec oxidase (COX) and AOX are all involved in nitrite to NO^•^ reduction, although a clear mechanism is established only for cytochrome oxidase under hypoxia. However, this may become increasingly important as partial pressures of oxygen are reduced from the ambient level (Gupta and Igamberdiev, 2011).

Nitric oxide can react immediately with superoxide originated from ETC, to form peroxynitrite (ONOO^-^). Through this reaction, superoxide probably plays a role in regulating free NO^•^ level (Leitner et al., 2009). The protonated form of ONOO^-^, the peroxynitrous acid ONOOH (pKa 6.8) is involved in many deleterious reactions, such as oxidation of DNA, lipids: protein thiols and iron clusters (Vandelle and Delledonne, 2011). Paradoxically, in systems where the toxicity comes predominantly from more toxic molecules such as peroxides, NO^•^ may elicit protective activity against them (Van Breusegem et al., 2001).

## REDOX REGULATION IS AN ESSENTIAL FEATURE OF PLANT MITOCHONDRIAL FUNCTION

Mitochondrial ROS generation can be perpetuated throughout a broad number of reactions yielding different reactive species that serve as substrates for the specific antioxidant enzymes. The mitochondrial antioxidant system, through superoxide and peroxides detoxification, has a pivotal role affecting redox signaling.

### Mn-SOD AND ENZYMES OF THE ASCORBATE-GLUTATHIONE CYCLE

#### Mn-SOD

**FIGURE 1:**
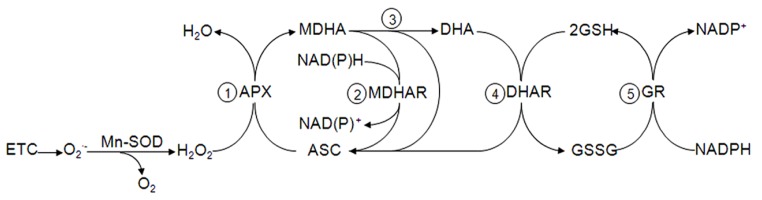
**Mitochondrial ascorbate-glutathione cycle.** The hydrogen peroxide in the mitochondria produced by ETC is reduced by APX at the expense of ASC to produce MDHA (step 1) that is either reduced to ASC (step 2) or disproportionated to DHA and ASC (step 3). DHAR reduces DHA using GSH as electron donor (step 4), which is regenerated by GR and NADPH (step 5).

In plants, Mn-SOD (**Figure 1**) appears as a tetrameric isoenzyme initially purified and characterized in *Pisum sativum* leaves (Sevilla et al., 1982), and located in both, mitochondria and peroxisomes (del Río et al., 1992). Numerous proteins have been identified as being dual targeted, mainly to plastids and mitochondria although around ten-twelve have been described as nuclear and plastidial, or mitochondrial and peroxisomal as Mn-SOD (Duchêne and Giegé, 2012). Mitochondrial and peroxisomal Mn-SOD expression is regulated differently in processes like leaf senescence, where post-translational events may regulate the enzymatic activity of the peroxisomal enzyme (del Río et al., 2003; Palma et al., 2006). Mn-SOD is important in providing protection against oxidative stress in these organelles, so avoiding the formation of more dangerous ^•^OH radicals and controlling H_2_O_2_ production. Defects in mitochondrial function are associated to a large number of different phenotypes. It has been reported that the lack of mitochondrial SODs in *Caenorhabditis elegans* mutants, in contrast to that reported in yeast or animals (Kirby et al., 2002), reduces not longevity but growth (Van Raamsdonk and Hekimi, 2009). In this case, a reduction in the metabolic energy observed could afford different explanations like the reported induction of uncoupling mechanisms, which reduced ROS generation in mitochondria, the decrease of the membrane potential and/or activity of the ETC. A similar reduction in growth has been described for Mn-SOD mutants in plants; in this case the respiration rate was not affected but the mitochondrial redox balance and some of the tricarboxylic acid (TCA) cycle enzymes were altered. Unexpectedly, Mn-SOD mutants displayed an increased antioxidant capacity, suggesting the existence of a retrograde pathway trying to compensate the lack of this antioxidant enzyme (Morgan et al., 2008). Reduction in growth is a general phenotypic characteristic in mitochondrial dysfunction and it may exhibit the interconnection established between mitochondrial metabolism and photosynthetic carbon assimilation. A complementary hypothesis has adduced the crosstalk between redox signaling and hormonal pathways regulating growth inhibition (Schwarzländer and Finkemeier, 2013).

#### ASC-GSH cycle

As a result of the $O_{2}^{\cdot -}$ dismutation, the newly formed H_2_O_2_can be decomposed by the mitochondrial peroxidase activities dependent on the antioxidants: (I) ascorbate (ASC) for the hemo-containing enzyme ascorbate peroxidase (APX; **Figure 1**), (II) the thiol reductant glutathione (GSH) for the glutathione peroxidases (GPX) and (III) the thioredoxin/peroxiredoxin system (Trx/Prx). The generated oxidized forms of ASC are then reduced by the FAD-containing monodehydroascorbate reductase (MDHAR) in an NAD(P)H-dependent manner and dehydroascorbate reductase (DHAR) using GSH as electron donor. Oxidized GSSG is reduced by the flavoprotein glutathione reductase (GR) and oxidized by thioredoxin reductase (NTR), both in an NADPH-dependent manner (Noctor and Foyer, 1998; Barranco-Medina et al., 2007; Martí et al., 2009). Accordingly, the antioxidant and redox systems in mitochondria depend on an adequate supply of NAD(P)H that is maintained by transhydrogenases in the mitochondrial membrane, as well as the enzymes isocitrate dehydrogenase and malate dehydrogenase in the matrix (Rasmusson and Moller, 1991).

The first publications reporting the presence of the some components of the so-called ASC-GSH cycle in mitochondria (**Figure 1**) appeared in 1981 and 1990 with MDHAR and GR of potato and pea mitochondria, respectively (Arrigoni et al., 1981; Edwards et al., 1990). The final proof of principle of a complete cycle in plant mitochondria, similar to that in chloroplast (Foyer and Halliwell, 1976), was later described in pea leaves (Jiménez et al., 1997). Using enzymatic latency assays, APX activity was located outside of the inner mitochondrial membrane whereas MDHAR was highly latent in intact mitochondria and was membrane-bound. These findings suggested that the electron acceptor and donor sites of this redox protein are not on the external side of the mitochondrial membrane. DHAR and GR were found in the mitochondrial matrix and the antioxidants ASC and GSH were present as demonstrated by chromatographic techniques. Biochemical data also indicated that the mitochondrial APX activity resulted in at least two isoezymes with different substrate specificity and sensibility to inhibitors when compared to that found in peroxisomes and chloroplasts (Jiménez et al., 1998). The possible presence of the isoenzymes linked to the inner face of the external membrane was described by Chew et al. (2003). The membrane location of APX and MDHAR suggested a dual complementary function for both enzymes: they could reoxidize endogenous NADH to maintain a constant supply of NAD^+^ for mitochondrial metabolism (Douce et al., 2001) and protection against H_2_O_2_ (del Río et al., 1998; Chew et al., 2003). Thus, both enzymes also contribute to the signal transduction processes that lead to specific gene expression by regulating the mitochondrial and cytosolic concentration of the diffusible signaling molecule H_2_O_2_ (del Río et al., 1996). The presence of the ASC-GSH cycle in nitrogen-fixing legumes root nodules has been proved as well as its protective activity toward mitochondrial-derived radicals in sensitive spots like the hemo o leghemoglobin groups (Puppo et al., 2005).

Pea GR and *Arabidopsis* MDHAR were described as dual-targeted proteins in plant cells (Creissen et al., 1995; Obara et al., 2002). In *Arabidopsis*, two genes encode GR, called GR1 encoding a cytosolic and peroxisomal protein, and GR2, found in chloroplast and mitochondria, which is lethal when it is inactivated at an early stage of embryo formation (Meyer et al., 2012). Using biochemical, targeting and proteomic assays, the presence of the ASC-GSH cycle was corroborated in mitochondria of *Arabidopsis* by Chew et al. (2003). These authors proposed an integrative coordination between chloroplast and mitochondria through the dual targeting of proteins such as APX, MDHAR, and GR gene products to both organelles, while DHAR only had a mitochondrial localization. It was postulated that the coordination between plastids and mitochondria might occur by the dual targeting rather than subtle retrograde signaling (Millar et al., 2001; Chew et al., 2003).

The presence of one isozyme of APX on the intermembrane space side of the inner membrane is convenient for the use of the ASC generated in this location. ASC is produced by the terminal enzyme L-galactono-1,4-lactone dehydrogenase (GalLDH) also attached to the inner membrane and located in the mitochondrial complex I, and its presence is required for the stability of the complex (Pineau et al., 2008). GalLDH activity is highly dependent on the availability of oxidized cytochrome c from the mitochondrial respiratory chain and is also regulated by redox controls such as glutathionylation (Bartoli et al., 2000; Millar et al., 2003; Leferink et al., 2009). In addition to the reductive GSH dependent DHA reduction, ASC regeneration may also be attributed to the respiratory ETC (Szarka et al., 2007) or linked to other redox compounds as glutaredoxin (Grx) and Trx systems (Potters et al., 2002; Meyer et al., 2012).

Levels and redox state of ASC have been shown to be involved in the modulation of photosynthesis by mitochondrial metabolism and a complementation has been suggested between AOX pathway and ASC to protect photosynthesis against photoinhibition. Respiration-dependent changes in mitochondrial ASC synthesis could regulate retrograde signaling as a common signal from both mitochondria and chloroplasts (Talla et al., 2011). A good example of such an inter-organelle communication is the ASC produced in the mitochondria and then transported into the apoplast. In contrast to GSH, ASC appears to exert its greatest influence by setting thresholds for apoplastic and cytoplasmic signaling (Munné-Bosch et al., 2013).

The second abundant antioxidant in plant tissues is the thiol compound GSH, participating in the detoxification of ROS, heavy metals and xenobiotics and in the cell cycle regulation (Rouhier et al., 2008; Foyer and Noctor, 2009; Diaz Vivancos et al., 2010). GSH is synthesized in plastids and cytosol and then transported to mitochondria, although the nature and regulation of these transporters is still unclear. The dicarboxylate/2-oxoglutarate transporter in the inner mitochondrial membrane has been proposed as a potential candidate, as reported in animals (Wilkins et al., 2012). Immunolabelling studies have proved the presence of GSH in both, mitochondria and chloroplast containing about 15–25% and 62–75% respectively of the total pool of GSH (Fernández-García et al., 2009).

Under non-stress conditions, GSH is presented mainly in its reduced form, but stress conditions and/or senescence and detoxification of ROS can lead to its oxidation impacting in the cellular redox state (Jiménez et al., 1998; Vanacker et al., 2006; Noctor et al., 2012). GSH is also emerging as a player in the intracellular redox potential regulation, protection and signaling through PTMs such as glutathionylation of specific target proteins (Zaffagnini et al., 2012b) involving Cys residues. A link between complex I (CI) activity and GSH has also been shown in CI *Arabidopsis* mutants insensitive to a GSH biosynthesis inhibitor and with higher levels of GSH, implying an as yet unexplained effect of mitochondrial respiration on GSH homeostasis (Koprivova et al., 2010).

### Mn-SOD, AOX AND ENZYMES OF THE ASCORBATE-GLUTATHIONE CYCLE IN STRESS RESPONSE AND SIGNALING

Abiotic stress can produce contradictory effects depending on the specie, tissue analyzed and the developmental stage of the plant. The plant acclimation also depends on the application time and strength of the treatment. Mitochondria are central organelles in setting cellular redox balance and homeostasis (Noctor et al., 2007). Increased ROS production in the mitochondria along with the antioxidant defense orchestrating the cellular stress response, including salinity, has been well documented (Hernández et al., 2000; Mittova et al., 2003; Taylor et al., 2009). ROS production in mitochondria has been reported to increase under salinity and drought conditions. A stimulation of $O_{2}^{\cdot -}$ generation dependent on NADH- and succinate has been reported in plants under salinity, with a higher increase in sensitive cultivars than in tolerant plants (Hernández et al., 1993; Pastore et al., 2007). Furthermore, oxidative damage induced by NaCl stress can affect different cellular targets selectively: complex I of the ETC was found to be damaged via oxidative stress while complex II directly by salt (Hamilton and Heckathorn, 2001). In this context, changes in ROS levels caused by the perturbation of the respiratory complex I: have been proposed to trigger a mitochondrial retrograde signal (Rhoads and Subbaiah, 2007).

The adaptive response of plants induced by salt stress is well documented; in *Arabidopsis*, of 300 salt stress-induced genes, more than half had a predicted mitochondrial localization (Heazlewood et al., 2007). In general, an induced expression of antioxidant defense genes is usually correlated with enhanced salt stress tolerance (Hernández et al., 2000; Attia et al., 2008) although the molecular mechanisms involved in the regulation of this induction remains unrevealed (Foyer and Noctor, 2005). Moreover, changes at a transcript level did not usually correlate well with changes in protein responsive to stress, and post-transcriptional mechanisms are believed to play an important role in defining the mitochondrial stress response (MSR; Van Aken et al., 2009b).

Alternative oxidase in one of the components of MSR and has been used as a model system to study MRR (Van Aken et al., 2009b). *Arabidopsis AOX1a* mutant plants have been described as exihibiting altered antioxidant transcripts of both chloroplasts and mitochondria, when exposed to a combination of drought and light stress (Filipou et al., 2011). Interestingly, the ABI4 transcription factor involved in the chloroplast-nucleus signaling is responsible for the transcriptional regulation of *AOX1a* (Giraud et al., 2009). Transcripts encoding AOX genes, mainly *AOX1a* and *AOX1d*, are highly responsive to stress including salinity. In fact, plants constitutively over-expressing *Ataox1a*, with increased AOX capacity, showed lower ROS formation and improved growth in salinity conditions (Smith et al., 2009). Yet, discrepancies in AOX expression and *in vivo* activity have also been reported, and recently discussed (Ribas-Carbó et al., 2005, Rasmusson et al., 2009). Overall, the current knowledge in AOX attributes it an important role in stress adaptation in plants while its participation in cell re-programming under salinity stress has been proposed (Clifton et al., 2006).

Mitochondrial Mn-SOD, has also been reported to modulate its expression in response to salinity stress (Kaminaka et al., 1999; Dai et al., 2009; Rubio et al., 2009), undergoing an overexpression in tolerant cultivars while decreasing in salt sensitive ones (Hernández et al., 2000). This seminal observation has gained additional support by the fact that the overexpression of Mn-SOD in transgenic *Arabidopsis*, poplar, rice and tomato plants showed increased salt tolerance (Tanaka et al., 1999; Wang et al., 2004, 2007, 2010a). The study of the changes in Mn-SOD protein revealed that, in tolerant pea plants, this protein was maintained with the duration of the salt treatment (Camejo et al., 2013a), while proteomic studies have shown that Mn-SOD of *Arabidopsis* accumulated during NaCl stress (Jiang et al., 2007). Also, mitochondria from salt-tolerant and salt-sensitive wheat cultivars subjected to salinity stress showed augmented levels of Mn-SOD and AOX, along with changes in cysteine synthase required for GSH formation. The coordinated increase in Mn-SOD and AOX proteins is thought to prevent the over-reduction of the mitochondrial ubiquinone pool, so lowering the content of superoxide in this organelle. The marked overexpression of these enzymatic systems responds to the specific adjustment of the cells in response to the oxidative stress. The fact that the vast majority of the non-redox proteome remained unchanged under saline stress strengthens this hypothesis (Jacoby et al., 2010). These authors suggest that the differences in proteomes of wheat varieties correlated with whole-plant salinity tolerance.

The heterogeneity of the antioxidant systems response under stress is manifested in numerous occasions. Each isoform of the same antioxidant enzyme in the different cell compartments can present a specific profile activity in lines/cultivars differing in salt tolerance (Olmos et al., 1994; Ashraf, 2009). A correlation between expression, protein and activity levels is usually found for Mn-SOD. Salt-tolerant tomato, pea and wheat cultivars have shown higher activity of mitochondrial Mn-SOD compared with a salt-sensitive cultivar (Hernández et al., 1993; Sairam and Srivastava, 2002; Mittova et al., 2003). This is not the case of peroxisomal Mn-SOD isoform since it was not induced in response to salt stress either in the tolerant or in the sensitive pea plants (Corpas et al., 1993).

As previously noted, GSH and ASC have a strong influence in gene expression (Munné-Bosch et al., 2013). The balance of reduced to oxidized forms of both antioxidants is crucial for the cell to sense oxidative stress and to respond accordingly (Mullineaux and Rausch, 2005; Foyer and Noctor, 2009, 2011). Consequently, the ASC/GSH pathway plays an essential role to cope the oxidative stress imposed by environmental stress including salinity (Hernández et al., 2000, 2001; Pallanca and Smirnoff, 2000; Gómez et al., 2004; Sharma and Dubey, 2005; Hefny and Abdel-Kader, 2009; Noctor et al., 2012). The existence of balance mechanisms to maintain ASC and/or GSH-dependent processes and related signaling response in specific compartments, when their respective contents are depleted, has been well established (Foyer and Noctor, 2011). In contrast, information on mitochondrial ASC and GSH contents and redox state is scarcely reported and their accurate role in mitochondria under abiotic stress is not well stated.

Information on the enzymes responsible to maintain and regulate the reduced/oxidized state of mitochondrial ASC and GSH, shows that the regulation of their gene expression presents high plasticity, and is an important component in the response of plants to stressful conditions.

The expression of *APX* encoding genes is modulated by various environmental stimuli, such as drought and salt (Hernández et al., 2000; Menezes-Benavente et al., 2004; Gill and Tuteja, 2010; Bonifacio et al., 2011). Very scarce information has been published on the APX mitochondrial isoform. In mitochondria from *Oriza sativa*, Δ*OsAPX6* expression remained unchanged against salt stress (Teixeira et al., 2006), while other works have reported an induction for the same isoenzyme in rice (Yamane et al., 2010). The discrepancy in regulation for this and other *APX* genes might be due to the absence of standardized conditions of measurements, since each group used different cultivars, organs, plant age and growth conditions which, as related above, have an important contribution in plant stress response. The beneficial effects of APX have been documented in plants overexpressing this enzyme in chloroplast, peroxisomes and cytosol displaying an enhanced plant tolerance to salt and water deficit and ameliorating induced-oxidative injury (Badawi et al., 2004; Lu et al., 2007; Wei-Feng et al., 2008). A compensatory mechanism in rice mutant double silenced for cytosolic APXs by other antioxidant enzymes has been described, making the mutants able to cope with salt, heat, high light and methyl viologen stress, similar to non-transformed plants (Bonifacio et al., 2011).

Enzyme activity comparisons have proved that mitochondrial APX and GR are constitutively higher in salt-tolerant wheat cultivar than in sensitive plants although none responded to salinity (Sairam and Srivastava, 2002). This response was different in mitochondria from tolerant pea plants, in which APX and MDHAR activities appeared early increased at mild salt stress and progressively increased under high salt concentrations, whereas GR and Mn-SOD were induced only after severe salinity. In chloroplasts and peroxisomes, these isoenzymes responded differently than in mitochondria, although stromatic APX, but not thylakoidal, was significantly and progressively increased, together with DHAR in response to the severity of the salt stress (Corpas et al., 1993; Gómez et al., 1999, 2004). The study in tomato revealed a decreased oxidative stress in a tolerant salt cultivar which, in part, was attributed to induced activities of Mn-SOD and mitochondrial APX, as previously commented in pea, as well as to increases of both ASC and GSH content in mitochondria, by a yet-unexplained mechanism (Mittova et al., 2004). Scarce information exists on the possible relation of these activities with the mitochondrial MDHAR and DHAR expression. Nonetheless, a compensative overexpression of different cytosolic and chloroplastic MDHAR and DHAR can enhance plant tolerance against various abiotic stresses (Gill and Tuteja, 2010) including salinity in tobacco, potato and *Arabidopsis* (Eltayeb et al., 2007, 2011; Wang et al., 2010b).

Similarly, the regulation of the GR has been proved to efficiently respond to different stresses (Creissen et al., 1994). A cytosolic GR gene was found induced in a pea salt tolerant, but not in the salt-sensitive, cultivar (Hernández et al., 2000) and the induction of the symplastic GR activity was higher in the tolerant plants, at the same time as increased DHAR and MDHAR activities. A putative role for all these enzymes in the control of symplastic/apoplastic ASC content was described (Hernández et al., 2001). The overexpression of GR has been shown to improve tolerance to oxidative stress, leading in tobacco and poplar to a higher ASC content in leaves (Aono et al., 1993; Foyer et al., 1995).

All together, these results suggest a fine-tuning for chloroplasts and mitochondrial signaling mechanisms to coordinate the response of these antioxidant enzymes for the acclimation of plants to salinity conditions.

### Trx/Prx/Srx SYSTEM

#### Thioredoxins

**FIGURE 2:**
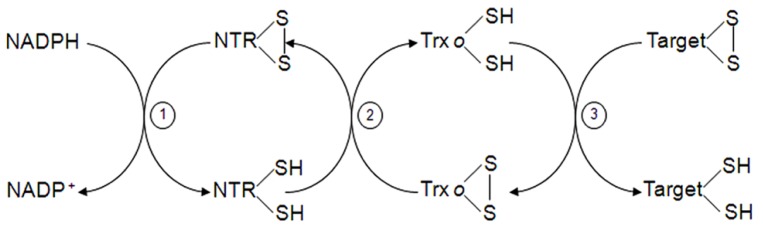
**Trx system in mitochondria.** Mitochondrial Trx*o* is reduced by NADPH-dependent TR (steps 1 and 2). Reduced Trx*o* can reduce in turn mitochondrial target proteins (step 3).

Thioredoxins (Trxs) are ubiquitous small proteins involved in the reduction of disulphide bonds of other proteins through a dithiol-disulfide exchange. They have a conserved active site WCG/PPC with reductive properties to regulate specifically target proteins. Plants, unlike bacteria and animals, contain several nuclear encoded *Trx* genes. In *Arabidopsis thaliana*, at least 20 *Trx* genes have been reported with different location (Collin et al., 2004; Meyer et al., 2012). The presence of Trx in plant mitochondria was shown by Laloi et al. (2001) in *Arabidopsis*, and was classified as Trx*o* type (AtTrx*o*1), although an additional mitochondrial *h*-type Trx was also localized in poplar (Gelhaye et al., 2004). More recently, a pea Trx*o*1 was described in both mitochondria and nucleus under normal conditions (Martí et al., 2009) while the localization of a nuclear Trx type *h* had been shown only under oxidative conditions in germinating wheat seeds (Serrato et al., 2001; Serrato and Cejudo, 2003; Pulido et al., 2009). Mitochondrial and cytosolic Trxs are reduced by a homodimeric FAD-NTR that utilizes NADPH (**Figure 2**), while chloroplastic ones use a ferredoxin-NTR system (Gelhaye et al., 2005). Two genes encoding NTR have been found in *Arabidopsis*: *AtNTRB*, which expresses the mitochondrial form and *AtNTRA*, expressing the cytosolic one (Reichheld et al., 2005; Tovar-Méndez et al., 2011). A new NADPH NTR (NTRC) has been demonstrated to exist in chloroplasts and non-photosynthetic plastids (Serrato et al., 2004; Kirchsteiger et al., 2012). NTRC have both, NTR and Trx, domains in the same polypeptide chain and reduces chloroplast 2-Cys Prx without the assistance of Trx (Pérez-Ruiz et al., 2006; Pulido et al., 2010). As far as we know, the high abundance of different Trx types in the cell as well as the redundant coexistence of different Trxs within the same organelle may reflect the presence of differential redox pathways for each. The specific function, protein–protein interaction and redox-network implication for the cited Trxs is far from being elucidated. Moreover, the high diversity in plants Trxs when compared with humans might add an additional antioxidant support in plants.

Although the extensive research in the last two decades has revealed diverse aspects of Trxs in plants, very little is known about the mitochondrial Trx function. It has been suggested that it is related to mitochondrial redox regulation and AOX (Balmer et al., 2004; Gelhaye et al., 2004; Martí et al., 2009; Yoshida et al., 2013) and, the detoxification of ROS via a mitochondrial PrxIIF has also been proposed (Barranco-Medina et al., 2008b). Application of the mutant affinity column approach by using cytoplasmic or chloroplastic forms of mutated Trxs, led to a systematic screening of Trx targets and thus, Balmer et al. (2004) were able to identify 50 potential Trx targets in mitochondria that covered major metabolic pathways. However, mutant PsTrx*o*1C37S in a proteomic assay with pea mitochondria only identified nine potential PsTrx*o*1 targets (Martí et al., 2009). Among the PsTrx*o*1-linked proteins there are components of the glycine decarboxylase complex and serine hydroxymethyl transferase (SHMT), key enzymes in photorespiration, and the alpha-subunit of the mitochondrial ATP synthase, which links Trx*o*1 with the control of ATP synthesis. Besides, the elongation factor Tu, that promotes the GTP-dependent binding of aminoacyl-tRNA to the ribosome, thiosulfate sulfurtransferase, mercaptopyruvate sulfurtransferase involved in sulfur metabolism and the drought stress related short-chain alcohol dehydrogenase were also identified.

Biochemical characterizations have reported that PsTrx*o*1 is able to activate two additional enzymes, the antioxidant PrxIIF (see below) and the respiratory enzyme AOX (Martí et al., 2009). Recently, Yoshida et al. (2013), using a similar methodology have found 101 Trx targets in mitochondria. Among them, the enzymes cited before have also been reported. A more detailed confirmation analysis by additional approaches is required to evaluate all these proteins as “true” targets, helping to understand the *in situ* functional significance of these Trx–target interactions.

Alternative oxidase has been identified in *Arabidopsis* as a protein of the inner mitochondrial membrane with an intramolecular disulfide bond (Winger et al., 2007). This protein is encoded by a small gene family, whose members have been shown to be both tissue-and development specific. AOX has not been identified as a Trx target using Trx-linked resins, although it can be both reduced and activated by mitochondrial thioredoxin PtTrx*h*2 by using its effector pyruvate (Gelhaye et al., 2004; Umbach et al., 2006). Similarly, PsTrx*o*1 specifically reduced pea mitochondrial AOX homodimers and produced the activation of oxygen consumption by this AOX pathway, using a NADPH/NTR system (Martí et al., 2009). Our comparative study of the published literature reveals the higher ability of PsTrx*o*1 to activate AOX in pea mitochondria compared with that in soybean organelle, presenting NADPH/NTR/PsTrx*o*1 as a highly effective system in the activation of the AOX pathway in pea. As reported, AOX plays an important role in preventing or minimizing ROS formation in cells (Maxwell et al., 1999; Yip and Vanlerberghe, 2001; Millar et al., 2011). Thus, we hypothesize that Trx*o*1, through the control of the reduced levels of AOX, might regulate respiratory metabolism and associated reactions. Trx*o*1 through activation of PrxIIF and AOX, could also play a role in linking ROS and redox signaling in mitochondria.

Several proteins have a dual localization to mitochondria and nucleus (Duchêne and Giegé, 2012) and a signaling function for mitochondrial biogenesis has been speculated. In pea leaves, PsTrx*o*1 was also found in nuclei with an apparent molecular mass of 20.6 kDa, corresponding to the protein translated and driven to the nuclei without the removal of the mitochondrial N-terminal targeting signal (Martí et al., 2009). Some plant Trxs have been found in the nucleus under stress conditions, i.e., Trx h typically located in the cytosol, has been reported to accumulate in the nucleus of aleurone and scutellum cells during germination (Serrato and Cejudo, 2003; Pulido et al., 2009). The function of PsTrx*o*1 in the nucleus is unknown although could be related to transcriptional regulation through oxidation protection of heterochromatin as proposed for the mammalian PRDX5 (Kropotov et al., 2006), the regulation of activity of several transcription factors (Hirota et al., 1997) and/or the control of apoptosis signal-regulated kinase 1 activity (Saitoh et al., 1998). Further studies seeking to identify functional targets for PsTrx*o*1 in the nucleus are needed to learn more about new physiological roles of this Trx*o*1 in plant cell.

#### Peroxiredoxins

Peroxiredoxins (Prxs) are thiol-based peroxidases involved in peroxide detoxification and play an important role in signaling (Wood et al., 2003). Prxs share a common catalytic mechanism where, by reducing peroxide, the catalytic active site Cys is oxidized to a sulfenic acid, which then forms a disulphide bond with a resolving Cys that is reduced by the Trx-NTR and NADPH system. Prxs reduce hydrogen peroxide and alkyl hydroperoxides to water and the corresponding alcohol, respectively. They were initially identified in yeast (Kim et al., 1988) and then in mammals and humans, with six different human Prxs (PrxI-VI) grouped in three types (Chae et al., 1994). The presence of plant Prxs was first discovered by Baier and Dietz (1996) and their classification does not correspond with the nomenclature established for human Prxs. Plant Prxs are divided into four subgroups based on the number and position of the conserved cysteine residues, namely 2-Cys Prx, type II Prx, Prx Q, and 1-Cys Prx, with different subcellular locations.

Type II Prxs are dimeric enzymes with varying molecular mass, isoelectric points and subcellular localization and have been proposed as primary sensors for hydrogen peroxide (Rhee et al., 2005). They were discovered in mammalian as a type of Prxs that forms an intramolecular disulfide as a reaction intermediate. In mammals, only one type II Prx (Prx V), with mitochondrial localization, has been found (Seo et al., 2000). In plants, three type II Prxs have a cytosolic (PrxIIB, C and D), one a chloroplastic (PrxIIE) and one a mitochondrial localization (PrxIIF; Horling et al., 2002). Plant mitochondrial PrxIIF is highly conserved between different species and contains the two cysteine-residues characteristic of type II Prx at positions 59 (peroxidatic Cys) and 84 (resolving Cys) of the mature protein (Finkemeier et al., 2005; Barranco-Medina et al., 2007).

**FIGURE 3:**
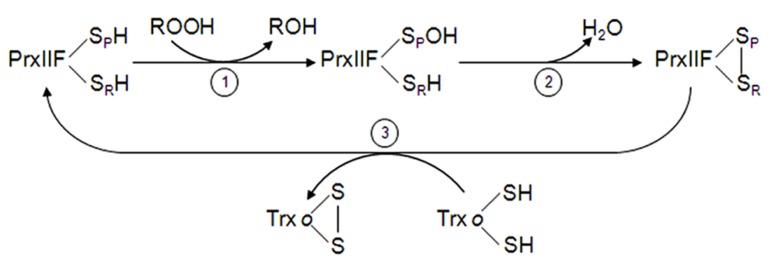
**Reaction mechanism of mitochondrial PrxIIF.** PrxIIF is oxidized to its sulfenic form in the reduction of peroxides (step 1). PrxIIF-SOH forms an intramolecular disulfide bridge (step 2) that is reduced by the mitochondrial Trx*o* system (step 3).

While the disulfide bridge formed in typical 2-Cys Prx, after hydroperoxide reduction, is intermolecular, in atypical type II Prx it is intramolecular (Seo et al., 2000). The catalytic cycle of PrxIIF consists of three steps (**Figure 3**): (1) the nucleophilic attack of the peroxide by the conserved peroxidatic Cys (Cys-S_P_H) that is oxidized to sulfenic acid (Cys-S_P_OH), (2) the formation of the disulfide by attack of the free thiol of the resolving Cys (Cys-S_R_H) to release water, and (3) the regeneration of the thiol form by mitochondrial Trx*o,* Grx and GSH as electron donors (Finkemeier et al., 2005; Gama et al., 2007; Barranco-Medina et al., 2008b). Rouhier et al. (2002) proposed a reaction mechanism for a cytosolic type II Prx from poplar in which only one of the two cysteinyl residues is involved in catalysis. Furthermore, Barranco-Medina et al. (2007), using two mutated variants, demonstrated that both Cys residues are essential for efficient catalysis. The interaction between Trxo and PrxIIF has been demonstrated with recombinant proteins and using C36S Trx*o* variant (Barranco-Medina et al., 2008b; Martí et al., 2009). The catalytic efficiency of plant PrxIIF (Rouhier and Jacquot, 2005; Barranco-Medina et al., 2007) is significantly higher than of 2-Cys Prx (König et al., 2003; Bernier-Villamor et al., 2004).

Structural studies of atypical Prxs have shown that PrxIIF dimerizes like typical 2-Cys Prx, but its dimerization is based on A-type, instead of B-type, interfaces (Echalier et al., 2005; Karplus and Hall, 2007). Moreover, the presence of high molecular weight species has been established (Evrard et al., 2004). Unlike 2-Cys Prx, that occurs as decamers, pea mitochondrial PrxIIF crystallizes as hexamers (Barranco-Medina et al., 2006) which are favored in oxidant conditions, but dissociate to dimers upon reduction (Barranco-Medina et al., 2008b). The presence of peroxidatic Cys was critical for hexamer formation whereas substitution of resolving Cys did not impact the oligomeric pattern (Barranco-Medina et al., 2007). By analogy with the dimer-decamer transition of the typical 2-Cys Prx (König et al., 2002; Wood et al., 2003; Bernier-Villamor et al., 2004; Barranco-Medina et al., 2008a, 2009), the dimer-hexamer transition in atypical PrxIIF displays a functional switch that could be involved in signaling (Barranco-Medina et al., 2008b).

Mitochondrial PrxIIF was one of the last identified antioxidants to be discovered in this organelle with functions in the reduction of hydrogen peroxide, playing also a chaperone-like activity (Finkemeier et al., 2005; Barranco-Medina et al., 2008b). In spite of the fact that mitochondria are one of the major sites of ROS generation in plant cells, and in contrast to other cellular compartments, PrxIIF is the only Prx type present in mitochondria. Its comparable activity with other Prxs and the presence of other efficient antioxidants in mitochondria bear witness to the auxiliary function of PrxIIF as H_2_O_2_ scavenger (Finkemeier et al., 2005). The recently reported signaling/chaperone functions of PrxIIF are no longer trivial and deserve special attention.

Recently, the overoxidized form of PrxIIF has been shown to work as a non-transcriptional rhythmic marker. The circadian clock is an endogenous 24 h oscillator regulating many critical biological processes in plants. One of the key characteristics of the circadian clock is that it is buffered against temperature, maintaining an approximately 24 h rhythm over a broad physiological temperature range. The existence of overoxidized PrxIIF and its retroreductive sulfiredoxin (Srx) systems raises the question as to whether or not this plant mitochondrial antioxidant could work as circadian clock (O’Neill and Reddy, 2011). This feature might be crucial to plants growing in a constantly changing environment. This unaddressed hypothesis is a challenge to future investigations to elucidate new functions of plant Prxs.

#### Sulfiredoxins

**FIGURE 4:**
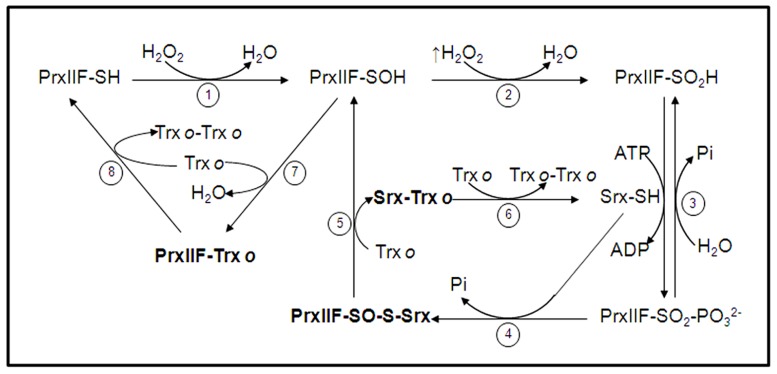
**Catalytic cycle of mitochondrial PrxIIF overoxidation and regeneration by Srx.** In physiological conditions mitochondrial PrxIIF is oxidized to its sulfenic form in the reduction of peroxides (step 1). At high concentration of H_2_O_2_ PrxIIF may be overoxidized to the inactive sulfinic form (PrxIIF-SO_2_H; step 2) that is phosphorilated, through a reversible step, in the presence of Srx and ATP (step 3). The phosphoril ester (PrxIIF-SO_2_-${PO}_{3}^{2-}$) is converted into sulfinate (PrxIIF-SO-S-Srx) with Srx and Pi is released (step 4). A reducing agent (mitochondrial Trx*o*) reduces the heterocomplex to release PrxIIF-SOH and Srx-Trx*o* (step 5). The complex Srx-Trx*o* is subsequently reduced to Srx-SH by Trx*o* (step 6). The sulfenic form of PrxIIF is reduced by Trx*o* that forms the intermolecular complex PrxIIF-Trx*o* (step 7) and the active PrxIIF-SH is released by another Trx*o* (step 8) that forms the dimer Trx*o*-Trx*o*. The binary complexes between the three proteins in the cycle (sulfinic PrxIIF, Srx and Trx*o*) are in bold type. (cif. ref. Iglesias-Baena et al., 2011).

Under oxidative conditions, Prxs undergo a transient oxidation of their cysteine residues from thiol to sulfenic acid and further stable disulfide bridges, which are regenerated to the thiolic forms by Trxs interaction (**Figure 4**). Under severe oxidative stress, Prxs rapidly overoxidize to the sulfinic (Cys-S_P_O_2_H) and sulfonic (Cys-S_p_O_3_H) form, locking the enzyme in a permanent inactive state which was primarily hypothethized to serve as an internal indicator of the hyperoxidative conditions inside the cells. The oxidation of the sulfenic acid to sulfinic acid was thought to be an irreversible step (Yang et al., 2002) until Woo et al. (2003) reported that the sulfinic form, produced under high levels of H_2_O_2_, was reduced to the catalytically active thiol form. These seminal observations served to suggest the presence of an enzyme able to retroreduce the overoxidized form of Prxs. These results were further confirmed by studies of different mammalian 2-Cys Prxs (Chevallet et al., 2003), but the identification of the proposed enzyme was carried out by Biteau et al. (2003). They observed how yeast treated with H_2_O_2_ induced overexpression of a new protein that they called Srx. Concurrently, deletion of the Srx gene reduced the tolerance of yeast to H_2_O_2_. Since its discovery in yeast (Biteau et al., 2003; Vivancos et al., 2005), Srxs have been studied, in mammals (Chang et al., 2004; Woo et al., 2005; Jeong et al., 2006), plants (Liu et al., 2006; Rey et al., 2007; Iglesias-Baena et al., 2010) and cyanobacteria (Pascual et al., 2010; Boileau et al., 2011).

The almost concomitant discovery of Srxs (Biteau et al., 2003) together with other redox active protein family called sestrins, described in prokaryote and animal systems (Budanov et al., 2004), as novel enzymes able to regenerate the overoxidized forms of Prxs brought several implications. Firstly, a new enzyme was added to the redox network of Prx adding a new level of regulation. Secondly, the span life of Prxs in the cell increased as a direct implication of their regeneration by Srx and sestrins. Consequently, the concept of a constant rate for “de novo Prx synthesis” needs to be reevaluated. Regeneration of Prxs partially challenges the idea of overoxidized Prxs as cellular indicators of overoxidation: assuming that only the sulfinic form of Prx can be retroreduced, only the sulfonic overoxidized form could work as permanent cell markers as long as they last inside the cell and before being degraded by the cell scavengers. The controversial capability of sestrins to retroreduce overoxidized Prxs (Woo et al., 2009) has drastically diluted their impact in the redox literature, while Srxs have emerged as their clear regenerators, establishing the triade Trx-Prx-Srx.

Sulfiredoxin are a special type of ATP-dependent reductase containing a conserved C-terminal cysteine critical for their antioxidant function (Jönsson and Lowther, 2007). Originally, Srxs were thought to be exclusively involved in the reduction of the sulfinic form of typical 2-Cys Prxs (Woo et al., 2005). Subsequent studies carried out by Iglesias-Baena et al. (2011) demonstrated a broader specificity toward the inactive sulfinic forms of atypical plant PrxIIF and atypical human PrxV. These encouraging results stimulate future investigations to establish a general mechanism of retroreduction for the broad diversity of plants Prxs, which could, presumably, respond to the Prx type as well as the subcellular localization. Although mammal Srx are cytosolic, the sulfinic form of mitochondrial human 2-Cys PrxIII can be reduced by hSrx (Woo et al., 2005). Recently, Noh et al. (2009) have reported the hSrx translocation from cytosol to mitochondria under oxidative stress to reduce overoxidized hPrxIII. These results reinforce the hypothesis of a general mechanism of Srx assisting in the regeneration of a broad battery of Prxs. However, the fine mechanism of chemiotaxis targeting Srx to different compartments as a response to the redox conditions needs to be addressed. An aggressive oxidative stress would lead to an increment in the protein concentration and detection in mitochondria. A different scenario have been reported in plants (pea and *Arabidopsis*) in which Srxs were found in chloroplasts and mitochondria regardless of the redox state (Liu et al., 2006; Rey et al., 2007; Iglesias-Baena et al., 2010, 2011). Additional works with Srxs and Prxs from different organisms are needed to tackle the ambiguous localization and substrate specificity of Srx.

The mitochondrial Srx retroreduces the inactive sulfinic form of atypical PrxIIF, employing a mechanism similar to that proposed for other Srxs (Jönsson et al., 2008). One oxygen atom on the sulphinic moiety of the oxidized PrxIIF functions as a nucleophile and attacks the γ-phosphate of ATP at the Srx to yield a sulphinic acid phosphoryl ester intermediate that is resolved by the nucleophilic attack of the Cys from the Srx (**Figure 4**). This mechanism involves two binary complexes, namely PrxIIF-Srx and Srx-Trx*o*. Only the sulfinic form of PrxIIF interacts with Srx (Iglesias-Baena et al., 2011). A secondary complex Srx-Trx has been isolated through formation of a mixed disulfide between Srx and C36STrx (Iglesias-Baena et al., 2011). Roussel et al. (2009) have also demonstrated that Trx forms an efficient complex with Srx. Both complexes, PrxIIF-Srx and Trx-Srx strengthen the proposed mechanism for sulfinic PrxIIF reduction by Srx.

*Arabidopsis* *srx* (AtSrx) gene codes for a protein bearing a transit peptide in the N-terminus with the characteristics of dual import to chloroplast and mitochondria (Pujol et al., 2007; Mitschke et al., 2009; Iglesias-Baena et al., 2010, 2011). The mature Srx has a catalytic cysteine (Cys72) involved in the activity. Plant Srxs have an additional non-catalytic cysteine (Cys88; Iglesias-Baena et al., 2010) and, similar to mammalian Srxs, display low efficiency as retroreducing enzymes (Jönsson and Lowther, 2007). Unlike human Srx, only able to retroreduce typical 2-Cys-Prx, AtSrx has a lower substrate specificity showing activity toward typical and atypical Prxs, in different cellular compartments and in different organisms. The concentration of AtSrx was estimated as 0.2% of the total chloroplast protein (Iglesias-Baena et al., 2010, 2011).

Systematic site-directed mutagenesis and molecular modeling suggest that plant Srx has special characteristics that differentiate it from its counterparts in humans (Iglesias-Baena et al., 2010). Although this singularity of plant Srx does not change its reaction mechanism, the structural differences with mammalian Srx can be related with a broad specificity, including atypical Prxs.

### Trx/Prx/Srx SYSTEM IN STRESS RESPONSE AND SIGNALING

The involvement of Trxs, Prxs and Srxs in plant tolerance to abiotic stress including salinity is not widely reported in the literature (Barranco-Medina et al., 2007; Pulido et al., 2009; Tripathi et al., 2009). The existing data have allowed the attribution to the Trx/Prx/Srx system of a redox sensing and signal transduction function (Rouhier and Jacquot, 2005) as well as its participation in the repair of oxidized proteins during environmental stress (Dos Santos and Rey, 2006). Leaf transcriptome results of salt-tolerant and salt-sensitive poplar, revealed that Trx members including chloroplast and cytosolic Trxs, displayed an inconsistent response to salt stress in the leaves, with the majority of the genes unchanged, whereas others showed up- or down-regulation under salinity conditions (Ding et al., 2010). Regarding mitochondrial Trx*o*1, an early induction in its gene expression at short salt treatments (five days at 150 mM NaCl) was described in pea leaves, pointing to an adaptive behavior. Under long salt stress (15 days 150 mM NaCl), a parallel increase in Trx*o*1 activity and protein levels were found with an unexpected down regulation of the gen (Martí et al., 2011). At this long stress, the induction of Trx*o*1 activity was correlated with the *in vivo* activity of the alternative pathway (AP) and with an increase in its capacity, reflecting the presence of the sustainable active form of AOX. PsTrx*o*1 could then have a role through the regeneration of oxidized AOX to the functional reduced enzyme. Under salt stress, increasing amounts and activity of *Trxo1* might correlate also with either, the higher demand to regenerate the oxidized PrxIIF in mitochondria, or the interactions with other target proteins such as those of the photorespiration (Martí et al., 2011). More substantial biological information could be derived from the comparison between the overexpression of Trx*o*1 with its mammalian analog Trx2. Higher amounts of mitochondrial mammalian Trx-2, have been correlated with protection against *t*-butylhydroperoxide and etoposide-induced apoptosis (Chen et al., 2002) and cells deficient in Trx-2 had increased ROS production and exacerbated apoptosis (Tanaka et al., 2002).

Studies on the response of plant PrxIIF toward abiotic stress describe this mitochondrial antioxidant as a constitutive or responsive gen depending on the plant species and stress situation. No changes were described in PrxIIF mRNA levels in *Arabidopsis* leaves under NaCl, H_2_O_2_, light or ozone treatments (Horling et al., 2002, 2003; Dietz et al., 2006). However, transcript and protein levels were up-regulated in *Arabidopsis* roots after cadmium treatment (Finkemeier et al., 2005), and in poplar leaves after exposure to chilling and water deficit (Gama et al., 2007). In pea plants exposed to salinity, cold and cadmium stress, an up-regulation in PrxIIF mRNA transcript and protein levels was reported in leaves, but not in roots (Barranco-Medina et al., 2007), and the most recent work on pea leaf PrxIIF regulation adds a new time-dependent variable; PrxIIF presented a biphasic response toward salt stress increasing its transcript level after five days of treatment and decreasing after 14 days (Martí et al., 2011). Strikingly, PrxIIF protein content remained constant throughout the salt treatment but a PTM was detected at long time (see below; Camejo et al., 2013a).

**FIGURE 5:**
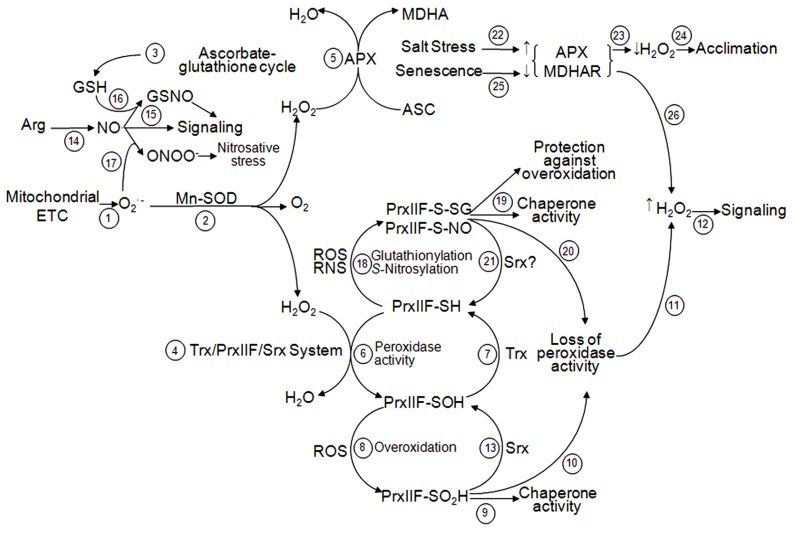
**Interaction between ascorbate–glutathione cycle and Trx/PrxIIF/Srx system in ROS and RNS signaling in plant mitochondria.** Superoxide radicals ($O_{2}^{\cdot -}$) produced by the mitochondrial ETC (step 1) are dismutated to molecular O_2_ and H_2_O_2_ by the activity of Mn-SOD (step 2). H_2_O_2_ is reduced by two different systems: ascorbate-glutathione cycle (step 3) and Trx/PrxIIF/Srx system (step 4). In the ascorbate-glutathione cycle, H_2_O_2_ is reduced by APX (step 5) and throughout the cycle as indicated in **Figure 1**. In the Trx/PrxIIF/Srx system, H_2_O_2_ is reduced by the peroxidase activity of PrxIIF (step 6) that produces its sulfenic form (PrxIIF-SOH) and is reduced by the Trx system (step 7) as indicated in **Figures 2 and 3**. Under oxidative stress, PrxIIF-SOH can be overoxidized to the sulfinic form (PrxIIF-SO_2_H; step 8) that gains chaperone activity (step 9) losing its peroxidase activity (step 10). This loss of activity increases H_2_O_2_ concentration in the mitochondria (step 11) allowing the signaling (step 12). PrxIIF-SO_2_H can be regenerated to the reduced form by the action of Srx (step 13) and Trx (step 7) as indicated in **Figure 4**. The generation of NO^•^ in the mitochondria (step 14) allows signaling (step 15) in addition of forming GSNO by reduction with GSH from ascorbate-glutathione cycle (step 16). NO^•^ can react with $O_{2}^{\cdot -}$, produced by the mitochondrial ETC, to form ONOO^-^ that bursts nitrosative stress (step 17). Under oxidative or nitrosative stress, PrxIIF-SH can be glutationylated or *S*-nitrosylated (step 18) in order to protect the enzyme against overoxidation and to gain chaperone activity (step 19). PrxIIF-S-SG and PrxIIF-S-SNO lose the peroxidase activity (step 20), allowing the signaling by H_2_O_2_ (step 11 and 12). These post-translational modifications could be reverted to PrxIIF-SH by the Srx activity (step 21) as it happens with the 2-Cys Prx. On the other hand, salt stress induces an increase of APX and MDHAR (step 22), which produces a decrease in the concentration of H_2_O_2_ (step 23) allowing acclimation (step 24). During the oxidative mechanism of senescence there are decreases of APX and MDHAR activities (step 25) which produce an increase in H_2_O_2_ (step 26) allowing signaling (step 12).

Although PrxIIF is involved in acclimation under salinity stress, the enzyme is not essential for plant survival. Lack of PrxIIF in knock-out lines of *Arabidopsis* does not worsen the cellular redox state under optimal conditions and its absence might be compensated by increased mitochondrial APX activity (Finkemeier et al., 2005) or by the presence of the ASC-GSH cycle in mitochondria (**Figure 5**). Notwithstanding the compensatory mechanisms, significant changes in expression of both nuclear and mitochondrial genes were described in the mutants, suggesting that, together with its antioxidant function, PrxIIF is an important candidate for perception of changes in the redox-state in the mitochondria (Finkemeier et al., 2005).

Regulation of Srxs under abiotic stress is not conclusive yet, in part due to their recent discovery and the few works addressing this topic. In *Arabidopsis*, an induction of the chloroplast Srx transcript level has been reported in plants responding to cold treatment (Liu et al., 2006) as observed with mitochondrial PrxIIF (Barranco-Medina et al., 2007). In *Arabidopsis*, the absence of Srx in knock-out lines (ΔAt*Srx*) produced an accumulation, not only of the inactive chloroplastic sulfinic form of 2-Cys Prx, but also of the mitochondrial sulfinic PrxIIF, which is in accordance with its dual location. Besides, the deletion of Srx yielded into more sensitive ΔAt*Srx* plants against high concentration of H_2_O_2_ when compared with AtWT plants (Iglesias-Baena et al., 2010, 2011). Although Srx is not essential for plant viability, it protects chloroplast and mitochondria depending on the intensity of the oxidative stress to regenerate the inactive sulfinic Prxs (**Figure 5**; Vivancos et al., 2005).

The sulfinic PrxIIF lacking of peroxidase activity can exhibit signaling functions in the cell. Therefore, Srxs, by controlling the reversion of the sulfinic form of PrxIIF, could indirectly regulate the signaling process (**Figure 5**). Herein, we propose an integrative model of signaling/antioxidant function taking into account ROS and antioxidants. On allowing H_2_O_2_ to carry out its signaling function, its level must increase rapidly above a threshold (Rhee, 2006). To maintain this concentration, some antioxidant enzymes must remain inactive; among them, mAPX and sulfinic PrxIIF could help in this aim. When the fast signaling function of H_2_O_2_ has finished, the peroxidase activities of mAPX and PrxIIF can be recovered. Furthermore, high levels of ROS in the mitochondria can lead to PrxIIF overoxidation contributing to its oligomerization and a switch of activity from peroxidase to chaperone (Barranco-Medina et al., 2006). The link between activity and oligomerization is well correlated and seems to establish a general mechanism for Prxs (see the review by Barranco-Medina et al., 2009).

All the reported changes of the antioxidant and redox systems imply that stress tolerance seems to require the induction of specific isoforms in the different cell compartments or a constitutively higher content of antioxidants, depending on the species, variety or strength and duration of stress. In this context, mitochondrial Mn-SOD, APX, MDHAR, AOX, Trx*o*1 and PrxIIF appear as key enzymes in the ROS network, functioning in both, salt adaptation and signaling pathways (**Figure 5**).

The miss-correlation existing between gene expression, protein level and activity evidences a complex regulation in the response of plants to changing environments, and points out the relevance of post-transcriptional and PTMs in the tolerance mechanisms involving MRR.

## POST-TRANSLATIONAL MODIFICATIONS: THE IMPORTANT ROLE OF *S*-NITROSYLATION IN MITOCHONDRIAL PROCESSES

### EFFECTS OF NO^•^ AND ITS DERIVATES ON MITOCHONDRIA

Mitochondria are exposed to NO^•^ on activation of enzymatic emission associated to ETC and arginine or diffused from surrounding cell compartments (del Río et al., 2002; Blokhina and Fagerstedt, 2010). NO^•^ can affect mitochondrial metabolism involving oxidation of metals in proteins complexes and reduction of free metal ions. NO^•^ can also react with oxygen to form oxidized NO^•^, which interacts with mitochondrial GSH to form *S*-nitrosoglutahione (GSNO; **Figure 5**) or with thiol-containing molecules to yield low molecular weight *S*-nitrosocysteine and *S*-nitrosocystein glicyne in a process called *S*-nitrosylation (Hess and Stamler, 2012). GSNO is considered to be the most abundant low-molecular mass *S*-nitrosothiol (SNO) and also a vehicle of NO^•^ throughout the cell, which enables NO^•^ activity to expand. GSNO has been located in pea mitochondria, together with cytosol, peroxisomes and chloroplasts (Corpas et al., 2013).

Prime targets of NO^•^ and its derivates in plants, are the mitochondrial electron transport components and enzymes, producing an inhibition of cytochrome c pathway whereas alternative respiration via AOX is only partially inhibited (Day et al., 1996; Martí et al., 2012). These inhibitions may potentially be involved in the regulation of energy metabolism, generation of ROS, cell death and response to stress. From a pharmacological approach, mitochondrial Mn-SOD has been found to be not inactivated by NO^•^ (Martí et al., 2012), although this enzyme binds and stimulates NO^•^ decay under both anaerobic and aerobic conditions (Filipovic et al., 2007). However, $O_{2}^{\cdot -}$ can react with NO^•^ three times faster than with mitochondrial Mn-SOD (Yamakura et al., 1998), so generating ONOO^-^ (**Figure 5**; Wilson et al., 2008), which represents a mechanism for NO^•^ consumption by mitochondria. Thus, when mitochondria respiration is inhibited by NO^•^, the formation of peroxynitrite contributes to NO^•^ degradation, reactivation of COX and restoration of oxygen consumption (Poderoso et al., 1996). The observed insensitivity of AOX to NO^•^also represents another mechanism to prevent its deleterious effects on respiratory activity (Millar and Day, 1996; Martí et al., 2012). Recent results highlight the importance of AOX in the control of NO^•^ level in plants; mitochondrial NO^•^ content is increased in the absence of AOX (Cvetkovska and Vanlerberghe, 2012), and curiously a NO-dependent up-regulation of *AOX* gene has been described (Huang et al., 2002). Furthermore the mitochondrial heme-enzyme APX (mAPX) was found to be reversibly inhibited by NO^•^in an ascorbate dependent manner, which could have physiological relevance during oxidative and/or nitrosative stress conditions where ASC depletion may occur (de Pinto et al., 2006; Martí et al., 2012). Hence, mAPX could be part of a NO^•^ redox signaling pathway in mitochondria, through the H_2_O_2_ and NO^•^ signaling cross-talk (Bright et al., 2006). Like Mn-SOD, mitochondrial MDHAR, DHAR and GR enzymes were not inhibited under continuous fluxes of NO^•^, which may contribute significantly to prevent a build-up of ROS, and also to allow the recycling of ASC and GSH from its oxidized forms, thus reducing the risk of RNS accumulation (Martí et al., 2012).

Overall the results probably indicate that not only NO^•^ resistant AOX but also mAPX, may be important components of the H_2_O_2_-signaling pathways under conditions inducing the production of NO^•^ in this organelle (Martí et al., 2012). New studies are needed to further elucidate the physiological relevance of the relation between mitochondrial NO^•^and derivates, and the ASC-GSH cycle enzymes under normal and stressful conditions.

### MITOCHONDRIAL TARGETS OF *S*-NITROSYLATION

Cellular functions of NO^•^ are carried out in part through *S*-nitrosylation, a dinamic PTM for the regulation of the protein function (Hess et al., 2005). Several mechanisms of *S*-nitrosylation have been proposed, but those operating in plant cells have not yet been elucidated (Kovacs and Lindermayr, 2013). Biological *S*-nitrosylation can take place by transnitrosylation, which involves the transfer of NO^•^ onto a cysteine thiol. Recent findings underscore the importance of subcellular compartimentation in determining when and where proteins are *S*-nitrosylated during signal transduction. In recent years, a long list of plant proteins undergoing *S*-nitrosylation and cellular processes affected has been identified from proteome-wide analysis by using NO^•^ donors as *S*-nitrosylating agents or by biotic and abiotic stressors, in cultured cells, whole leaves and cellular organelles (Lindermayr et al., 2005; Romero-Puertas et al., 2008; Astier et al., 2011; Fares et al., 2011). Thus, more than 50 *S*-nitrosylated candidate proteins were identified in *Arabidopsis* leaves, including cytosolic PrxIIB, chloroplast Trxf1 and a chloroplast PrxIIE, among others (Lindermayr et al., 2005). *S*-nitrosylation in PrxIIE was demonstrated to inhibit its peroxidase and peroxynitrite reductase activity. Authors suggested a model where this PTM regulates the transduction of NO^•^ and ROS-linked signals during infection by *P. syringae*, highlighting a key role for PrxIIE in controlling the endogenous level of ONOO^-^(Romero-Puertas et al., 2007, 2008). Recent studies have demonstrated that the Trx system may be involved in regulating the *S*-nitrosylation status of target proteins in different systems (Wu et al., 2011) through the transnitrosylation or denitrosylation, thus modulating their biological activities (Benhar et al., 2008; Kornberg et al., 2010). In mammalian systems, a subpopulation of caspase-3 in the mitochondria is constitutively *S*-nitrosylated and, as such, is inhibited, and mitochondrial Trx2 has been involved in this *S*-nitrosylation (Mitchell and Marletta, 2005), although the exact intramolecular mechanism of Trx denitrosylation remains ill-defined, as does the denitrosylation target specificity (Benhar et al., 2009). To the date, no information on the putative *S*-nitrosylation capacity for plant mitochondrial Trx*o*1 and its transnitrosylating/denitrosylating activity is available. In addition to Trx, *S*-nitrosoglutathione reductase (GSNOR) plays a predominant role in protein denitrosylation (Benhar et al., 2009). In mitochondria from *Arabidopsis* plants treated with GSNO, 11 proteins were identified as possible targets for *S*-nitrosylation (Lindermayr et al., 2005). Some of these proteins were mETC constituents and three of them were subunits of the glycine decarboxylase complex (GDC H1, T and P proteins), a key enzyme of the photorespiratory C2 cycle. The activity of this enzyme complex was inhibited by *S*-nitrosylation, and appears to be involved in the regulation of NO^•^ dependent signal transduction, although the underlying signaling pathway remains elusive (Palmieri et al., 2010).

It has been shown that AOX is co-expressed with GDC and the decrease of GDC amount in mitochondria also results in very low AOX levels (Bykova et al., 2005). GSNO treatment also inhibits complex I, and as a result, increases ROS in mitochondria, which could later affect chloroplastic ROS through reduced photorespiratory capacity (Gupta, 2011). Endogenous *S*-nitrosylation protein pattern in mitochondria from *Pisum sativum* leaves was reported as being similar or even higher than that found for mitochondrial proteins in other sources, including *Arabidopsis* (Tanou et al., 2009; Fares et al., 2011). A differential pattern of target proteins was identified during plant development, with a minor number of *S*-nitrosylated proteins in older plants, specifically some key enzymes related with respiration and photorespiration, including GDC T and GDC P subunits, NAD-MDH, succinate dehydrogenase, NADH ubiquinone oxidoreductase and ADP/ATP carrier, which disappeared as *S*-nitrosylated targets, while elongation factor Tu and protein kinase appeared as new targets of *S*-nitrosylation. Similar to GDC, *S*-nitrosylation of peroxisomal isozyme NAD-MDH also decreased its activity (Ortega-Galisteo et al., 2012). The differential *S*-nitrosylation pattern during development may be a mechanism for avoiding malfunction of the photorespiratory cycle and that of the respiratory components, which can further increase mitochondrial and chloroplastic ROS levels and affect redox signaling during this process (Camejo et al., 2013a). To date, the significance of the regulation of both processes by *S*-nitrosylation/denitrosylation during plant development remains undefined.

### *S*-NITROSYLATION UNDER SALT STRESS

The participation of NO^•^ in plants in response to biotic and abiotic stress including drought and salt stress has been demonstrated. The real role of NO^•^ in the cell is not exempt of controversy. While some authors consider NO^•^ as a stress-inducing agent, others have reported its protective task (Huang et al., 2002; Neill et al., 2003; del Río et al., 2004; Leitner et al., 2009; Rodríguez-Serrano et al., 2009). NO^•^ was recently proposed in the mediation of the response to salinity in different plant systems and varieties (Gould et al., 2003), not only for its harmful reactivity and toxicity but also for its involvement as signal molecule able to mitigate the damage associated with salt stress, either in plants and germinating seeds (Zheng et al., 2009). In pea plants, the increase observed in NO^•^ content by long-term salt treatments (14 days) was, in part, associated to mitochondria, although the contribution of other organelles, like peroxisomes and cytosol was not discarded (Rodríguez-Serrano et al., 2009; Camejo et al., 2013a).

In salt-stressed plants, the information for *S*-nitrosylation in mitochondria is quite limited, despite the significant developments in proteomics analysis allowing the identification of mitochondrial proteins suffering changes in abundance under salinity (Taylor et al., 2009; Jacoby et al., 2010). Recently 49 proteins including mitochondrial enzymes were identified as targets of *S*-nitrosylation in response to NaCl-stress in citrus plants (Tanou et al., 2009). In pea leaf mitochondria, at least 9 *S*-nitrosylated proteins were described under salt-stress, but as reported during pea development, as the stress became longer, so the number of identified protein targets decreased. This affected some enzymes related to NADH metabolism, as MDH, the photorespiratory GDC P and T subunit, and also Mn-SOD and aminomethyltransferase, identified as *S*-nitrosylated at short salinity period (5 days). Interestingly, mitochondrial PrxIIF and a heat shock Hsp90 protein appeared as new *S*-nitrosylation targets during the salt stress progression. Others enzymes involved in respiration were Succinate dehydrogenase, NADH ubiquinone oxidoreductase, and in photorespiration SHMT were found *S*-nitrosylated in control pea mitochondria but not under salt-stress. Thus, the denitrosylation of the respiratory activities may not limit ETC transport, at least through the AP which was not decreased with salt stress, as previously reported (Martí et al., 2011). The denitrosylation of the three key enzymes of photorespiration, was not accompanied by changes in its protein content and may allow this process to be functional after long period of salt stress. All these changes may contribute to the elevated NADH/NAD^+^ and the maintained NAD(P)H/NADP^+^produced in pea mitochondria inducing a modification in the mitochondrial matrix or ETC redox state. Several mitochondrial dehydrogenases are described to be affected by changes in the NADH/NAD^+^ ratio depending on their kinetics characteristics (Noctor et al., 2007). Another possible destination for reducing equivalents in mitochondria is the Trx system. In pea plants, the expression, content and activity of Trx*o*1 were increased under salt stress (Martí et al., 2011), so Trx*o*1 could play a pivotal role in sensing the local redox environment and regulating the activity of its target enzymes through reduction of their disulfide bridges. Consequently, functional photorespiration can prevent important photooxidative damage as results of the more intense salt-induced reduction of stomatal conductance at long-term salt stress (Martí et al., 2012). A functional role for the cooperation between the mitochondria, chloroplast and peroxisomes to modulate cell redox homeostasis under salinity and drought stress has been described (Noctor et al., 2007; Pastore et al., 2007).

The increase in NO^•^ content under salt conditions was not related to an enhanced mitochondrial protein *S*-nitrosylation (Camejo et al., 2013a), raising the question of whether NO^•^ could exhibit different actions. GSNOR activity was also induced under salt conditions and might constitute a mechanism of the degradation of SNOs, so influencing NO^•^ level in mitochondria by preventing NO^•^ kidnapping.

As a new target of *S*-nitrosylation under short salt stress, Mn-SOD does not seem to be affected in its activity, either under salt conditions or after GSNO treatment of mitochondria (Martí et al., 2012; Camejo et al., 2013a). These results corroborated the previously reported antioxidant function of this important enzyme under salt stress, as a ROS scavengers and possible NO^•^ sink (Hernández et al., 2000; Wang et al., 2004; Jacoby et al., 2010). Only during long-term salt stress, PrxIIF was found as *S*-nitrosylated (**Figure 5**) parallel to an increase of NO^•^, while the protein amount did not change. *S*-nitrosylation of PrxIIF was not previously described, although a strong posttranslational regulation to explain its response to high H_2_O_2_ concentration was suggested (Finkemeier et al., 2005). This modification may inhibit its peroxidase activity during salt stress, as indicated after treatment of the recombinant pea PrxIIF with GSNO (Camejo et al., 2013a). This inhibition suggests a role for PrxIIF as a signaling component more than as an antioxidative enzyme during long NaCl stress (**Figure 5**). Recently, a change in PrxIIF peroxidase activity to chaperone by *in vitro* *S*-nitrosylation of recombinant protein has been observed (Camejo et al., 2013b). It could be important to address whether this process is also taking place and to what intensity, under salt stress, when endogenous PsPrxIIF appears as *S*-nitrosylated.

## CONCLUSIONS AND PERSPECTIVES

Redox regulation and ROS metabolism are interlinked and involved in optimizing the function of cell organelles. The mitochondrial antioxidant system has a key role in the detoxification of $O_{2}^{\cdot -}$ and peroxides and thus plays a crucial role in controlling redox signaling. Redox proteome and *in vitro* recombinant protein studies have shown that many of the mitochondrial proteins undergo different redox PTMS, so modulating their antioxidant activity. A good example is Prx, including mitochondrial PrxIIF and Prx 3 in plants and mammalian, respectively. Thus, PrxIIF can be partially inactivated by hyperoxidation, glutathionylation and *S*-nitrosylation or by the extent of its oligomerization. To relate these modifications to events in situ it is important to distinguish between protective and signaling purposes under physiological and abiotic stress responses. Also, to understand how these redox PTMs regulate mitochondrial redox signaling it would be necessary to know how the different types of PTMs are coordinated and/or interconnected to allowing specific proteins to respond not only to different stimuli, but also to their intensity and duration.

Many thiols in proteins are susceptible to redox modifications, but only a few are important in signaling pathways. Some studies also highlight the concept that some protein cysteine residues are differently susceptible to NO-modifications and the functionality of reactive Cys as NO-sensor “*in vivo*” and their regulation by NO affecting biological processes is still little known. The study of these aspects would help to clarify the true significance of redox signaling.

Post-translational modifications on plant mitochondrial ASC-GSH cycle enzymes have not yet been studied in detail and although the reversible inhibition of APX by NO and its irreversible inactivation by ONOO^-^ have been reported, there is scarce information about the “*in vivo*” NO’s effects on these antioxidant enzymes. The link between NO^•^ and the ASC-GSH cycle enzymes is an essential target to clarify NO^•^ participation in redox signaling that awaits further functional characterization.

Similarly, the redox state of the Trx*o*1 can reversibly affect the activity of target proteins. Moreover, given that some Trxs can reduce sulfenic acids, SNOs and glutathionylated cysteines and could promote trans-nitrosylation reactions, the putative involvement of Trx*o*1 in some of these reactions should be analyzed in future studies. Additionally, the mechanism of protein deglutathionylation catalyzed by Srx still needs to be explored as well as its functional significance.

Thus, another interesting question will be to assess the functional aspect of AOX redox regulation by Trx*o*1 in controlling mitochondrial NO^•^ levels. This aspect may help to reinforce the model of cross-talk between NO/ROS in mitochondria. It would be also advisable to study the putative interaction between mAPX and Trx*o*1 for a better understanding of the mitochondrial response to oxidative stress.

Finally, an understanding of the above proposed aspects of the cross-talk between signaling pathways linked to ROS and RNS is also a major issue to elucidate the mechanisms underlining plant abiotic stress tolerance.

## Conflict of Interest Statement

The authors and the associate editor declare that no relationship exists between the editor and the authors. Although some of them have the same affiliation, they do not have any research or projects in common and they belong to different research groups. Moreover, this manuscript was conducted in the absence of any personal, professional, commercial or financial relationships that could be construed as a potential conflict of interest.
